# Bidirectional Longitudinal Associations Between Cognitive Abilities and Social Relationships in Old Age

**DOI:** 10.1093/geroni/igaa057.1915

**Published:** 2020-12-16

**Authors:** Minxia Luo, Peter Edelsbrunner, Jelena Siebert, Mike Martin, Damaris Aschwanden

**Affiliations:** 1 University of Zurich, Zurich, Zurich, Switzerland; 2 ETH Zurich, Zurich, Zurich, Switzerland; 3 Heidelberg University, Heidelberg, Baden-Wurttemberg, Germany; 4 Florida State University, Tallahassee, Florida, United States

## Abstract

Individuals’ social connections can both shape and be shaped by cognitive abilities in aging process. This study examined bidirectional longitudinal associations between cognitive abilities and social relationships using 12-year longitudinal data (3 waves) from 499 German older adults who were born between year 1930 and 1932. Cognitive abilities were assessed as a latent construct consisting of five cognitive tests, i.e., picture completion, block design, information, similarities, and word finding. Social relationships were assessed by the self-reported number of free time partners and scales of perceived social relationships. Using a random-intercept cross-lagged panel model, we focused on within-person causal relations. Results showed that higher cognitive abilities predicted higher number of free time partners over four years and that more positive perceived social relationships predicted higher cognitive abilities at four-year follow-up. In sum, the bidirectional longitudinal associations indicate social relationships and cognitive abilities mutually maintain each other in old age.

